# Bringing two worlds closer together: a critical analysis of an integrated approach to guideline development and quality assurance schemes

**DOI:** 10.1186/s12913-020-05819-w

**Published:** 2021-02-24

**Authors:** Thomas Piggott, Miranda Langendam, Elena Parmelli, Jan Adolfsson, Elie A. Akl, David Armstrong, Jeffrey Braithwaite, Romina Brignardello-Petersen, Jan Brozek, Jolanta Gore-Booth, Markus Follmann, Zbigniew Leś, Joerg J Meerpohl, Luciana Neamţiu, Monika Nothacker, Amir Qaseem, Paolo Giorgi Rossi, Zuleika Saz-Parkinson, Philip van der Wees, Holger J. Schünemann

**Affiliations:** 1 Department of Health Research Methods, Evidence, and Impact, McMaster University Health Sciences Centre, Room 2C16, 1280 Main Street West, Hamilton, ON L8N 4K1 Canada; 2 Department of Epidemiology and Data Science, Amsterdam UMC, University of Amsterdam, Amsterdam Public Health institute, Amsterdam, Netherlands; 3 European Commission, Joint Research Centre (JRC), Ispra, Via E. Fermi 2749 – TP 127, I-21027 Ispra, VA Italy; 4 Swedish Agency for Health Technology Assessment and Assessment of Social Services, Sweden & The Department of Clinical Science, Intervention and Technology, Karolinska Institutet, Stockholm, Sweden; 5 Department of Internal Medicine, American University of Beirut, Beirut, Lebanon; 6 Farncombe Family Digestive Health Research Institute, McMaster University, Hamilton, Canada; 7 Department of Medicine, McMaster University, Hamilton, Canada; 8 Australian Institute of Health Innovation, Macquarie University, Level 6, 75 Talavera Rd, Sydney, 2109 Australia; 9 Digestive Cancers Europe, Brussels, Belgium; 10 German Cancer Society, Heidelberg, Germany; 11 Evidence Prime, Kraków, Poland; 12 Institute for Evidence in Medicine, Medical Center and Faculty of Medicine, University of Freiburg, Freiburg, Germany; 13 Institute of Medical Knowledge Management, Association of the Scientific Medical Societies, Frankfurt, Germany; 14 American College of Physicians, Philadelphia, PA USA; 15 Azienda Unità Sanitaria Locale - IRCCS di Reggio Emilia, Reggio Emilia, Italy; 16 Radboud University Medical Center, Department of IQ healthcare and Rehabilitation, Nijmegen, The Netherlands; 17 The George Washington University, School of Medicine and Health Sciences, Department of Clinical Research & Leadership, Washington, D.C., USA

**Keywords:** Guidelines, Quality indicators, Healthcare quality, Recommendations, Quality assurance, Quality improvement, Tools

## Abstract

**Background:**

Although quality indicators are frequently derived from guidelines, there is a substantial gap in collaboration between the corresponding parties. To optimise workflow, guideline recommendations and quality assurance should be aligned methodologically and practically. Learning from the European Commission Initiative on Breast Cancer (ECIBC), our objective was to bring the key knowledge and most important considerations from both worlds together to inform European Commission future initiatives.

**Methods:**

We undertook several steps to address the problem. First, we conducted a feasibility study that included a survey, interviews and a review of manuals for an integrated guideline and quality assurance (QA) scheme that would support the European Commission. The feasibility study drew from an assessment of the ECIBC experience that followed commonly applied strategies leading to separation of the guideline and QA development processes. Secondly, we used results of a systematic review to inform our understanding of methodologies for integrating guideline and QA development. We then, in a third step, used the findings to prepare an evidence brief and identify key aspects of a methodological framework for integrating guidelines QA through meetings with key informants.

**Results:**

Seven key themes emerged to be taken into account for integrating guidelines and QA schemes: (1) evidence-based integrated guideline and QA frameworks are possible, (2) transparency is key in clearly documenting the source and rationale for quality indicators, (3) intellectual and financial interests should be declared and managed appropriately, (4) selection processes and criteria for quality indicators need further refinement, (5) clear guidance on retirement of quality indicators should be included, (6) risks of an integrated guideline and QA Group can be mitigated, and (7) an extension of the GIN-McMaster Guideline Development Checklist should incorporate QA considerations.

**Discussion:**

We concluded that the work of guideline and QA developers can be integrated under a common methodological framework and we provided key findings and recommendations. These two worlds, that are fundamental to improving health, can both benefit from integration.

## Background

Development of guidelines and quality assurance (QA) schemes in health traditionally operate in two different worlds despite the fact that they are both critical, interdependent health improvement processes, designed to ensure that the best possible health recommendations are developed and that the recommended interventions ultimately meet the specified quality standards. Too often, these worlds do not connect well resulting in the development of quality indicators that are not linked to recommendations from respective guidelines, or recommendations that are not easily translated into quality indicators [1, 2].

Guidelines are used by diverse organisations to provide recommendations to practitioners and policy-makers regarding healthcare and clinical decisions. Several respected institutions have developed standards for trustworthy guidelines [2–9] that address the importance of engaging multidisciplinary stakeholders, using systematic reviews of the evidence to support recommendations, describing subgroups and peoples’ values and preferences, managing conflicts of interest, rating certainty or quality of evidence, moving from evidence to recommendations transparently, and routinely updating guidelines. The Grading of Recommendations Assessment, Development and Evaluation (GRADE) working group has developed a “common, sensible and transparent approach to the grading of evidence … and is now considered the standard in guideline development” [10]. GRADE has provided guidance to improve the guideline development process in the form of a detailed guideline development checklist [4].

However, developing measurable targets based on recommendations and ensuring their implementation and evaluation through QA schemes are equally critical to assess progress towards the realisation of health benefits. We use the ISO definition of QA being “the part of quality management which is directed at the creation of trust that quality requirements are satisfied” [11]. To develop QA schemes, several organisations have produced guidance on QA standards. For example, the International Society for Quality in Healthcare (ISQua) has issued guidelines which includes six steps from the development of standards to the monitoring and evaluation of quality performance [12]. These steps relate to: standards development; standards measurement; organisation role, planning, and performance; safety and risk; patient and service; and quality performance. The Agency for Healthcare Research and Quality has also presented key questions to inform the development of quality indicators [13]. The Guidelines International Network (GIN) has developed reporting standards for guideline-based performance measures [14]. However, a recent systematic review suggests that there are very few examples of integrated approaches to guidelines and QA [15].

The European Commission’s Joint Research Centre (JRC) has a mandate to produce independent scientific advice to inform European Union policy. One recent priority topic for guideline and QA advice was breast cancer [16]. The European Commission Initiative on Breast Cancer (ECIBC) attempted to combine guideline development and QA in an integrated fashion. This process formed two groups, one for guidelines and one for the QA scheme, which operated simultaneously and independently, with topics relevant to QA being referred, as they arose to the QA scheme development group. Lessons learned from this large multi-year process suggested that future initiatives should consider an approach where guideline recommendations and QA schemes are developed in a more integrated fashion and that a single common expert group is preferable. The European Commission tasked a steering group to prepare a methodological approach that would integrate QA and guideline processes for the European Commission Initiative on Colorectal Cancer (ECICC) and other similar future initiatives [17].

This is the first in a series of three articles describing the results of this work. This work aimed to identify key issues and to provide solutions on the integration of guidelines and QA. These fields, two worlds, coming together have the potential to yield significant benefits to patients, providers, and health systems, through more effective implementation of guidelines and quality assurance to ultimately improve health outcomes. In this article, we focus on the approach we used to identify and analyse the potential challenges for the development of an integrated methodological framework for guidelines and QA, and specifically present the results of a risk analysis and key themes that emerged through the process. The second article describes the findings of a systematic review of integrated approaches to guideline development and QA which informed this work. The third article provides a draft framework and addresses overdue clarification of the confusing terminology that is used in this field [15, 18].

In this article, we focus on the approach we used to analyse the potential challenges for the development of an integrated methodological framework for guidelines and QA, and specifically present the results of a risk analysis and key themes that emerged through the process. This work aims to provide identify key issues on the integration of guidelines and QA. These fields, two worlds, coming together have the potential to yield significant benefits to patients, providers, and health systems, through more effective implementation of guidelines and quality assurance to ultimately improve health outcomes.

## Methods

**Fig. 1:**
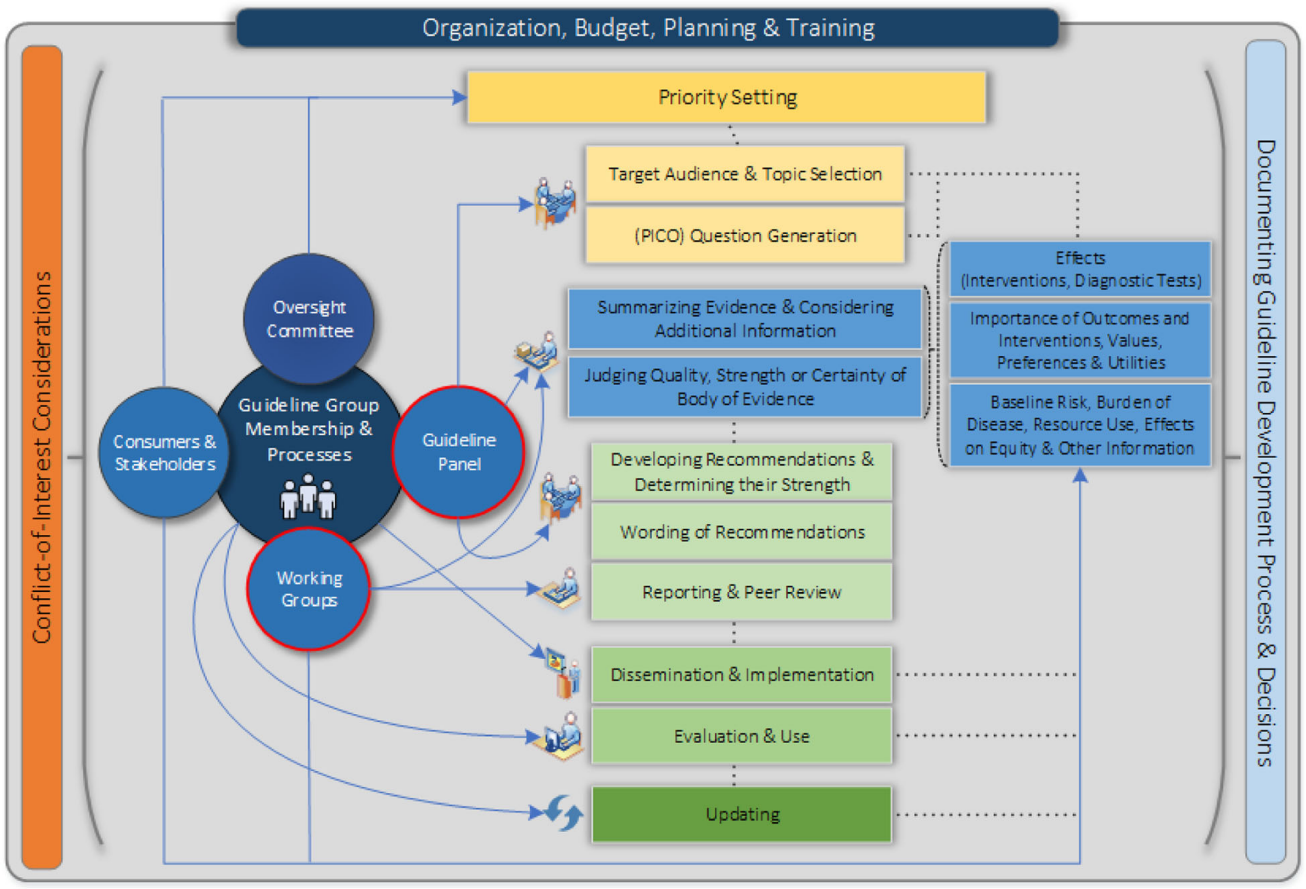
Integrated guideline and QA Framework Development Process. Figure 1 shows the steps leading to the development of the final methodological framework for an integrated guideline and QA scheme from the feasibility study, systematic review, evidence brief and workshop

We used a mixed-method approach incorporating findings from qualitative interviews, systematic review of the literature and stakeholder surveys. Figure 1 describes our stepwise mixed-method approach, which we elaborate upon in the methods sections that follow. Following work with the ECIBC, where QA and guideline processes were separate, we conducted a preliminary feasibility study to assess feasibility of pursuing an integrated approach to QA and guidelines. We updated an existing systematic review on methods to integrate guideline and quality assurance scheme [18]. These elements formed the basis for an evidence brief and to identify key aspects of a methodological framework for integrating guidelines and QA, through meetings with key informants. Discussions were then continued in multiple teleconferences. This work was assessed in December 2017 by the Hamilton Integrated Research Ethics Board as a quality improvement study and exempt from full research ethics review.

### Feasibility study

The feasibility study involved in-person and webinar meetings among the steering group. The steering group was composed of four researchers (HJS, TP, ML, EP), one from JRC and three from other organisations. All four have experience with health research methodologies and were involved in the ECIBC project. We first assessed and integrated feedback and observations from guideline, QA and JRC staff participants in the ongoing ECIBC. We also conducted key informant interviews with external experts (*n* = 10) purposefully sampled from different jurisdictions and organisations with guideline and QA scheme development expertise. Experts were selected from a convenience sample of expert who had been involved in the ECIBC project, experts known to the European Commission, and an internet search for academic experts involved in guideline and QA scheme development. In the interviews we discuss a strengths, weaknesses, opportunities, and threats (SWOT) analysis of an integrated guideline and QA scheme methodology. These interviews were conducted via GoToMeeting webconferencing (Citrix Systems, FL, USA) and were audio recorded and transcribed for thematic analysis.

### Systematic review

The findings from the feasibility study informed the development of the PICO (Population-Intervention-Comparison-Outcome) question for the systematic review. We then identified an existing systematic review from 2012 by Kötter et al. with a matching PICO question and therefore carried out an update of it [19]. The questions the review addressed were: 1. To identify and describe approaches that are utilized to develop guideline recommendations and quality assurance (QA) schemes (including quality indicators (QI) and performance indicators (PI)) in an integrated framework, i.e., development of guideline-based QA schemes; 2. To evaluate the effects of a guideline-based QA scheme on: Patient/individual health outcomes and processes; Structural outcomes: time required to develop recommendations and QA scheme; feasibility; acceptability by key stakeholders; and development costs. We present the methods and findings of this systematic review in a companion article [15].

### Evidence brief and draft methodological framework

We developed a detailed evidence brief as a background document for participants in the workshop [20]. To formulate the evidence brief, we synthesised findings from the systematic view, key informant interviews, and other seminal documents identified by key stakeholders through the feasibility study on guideline and QA scheme development and methodology.

We developed a draft methodological framework that outlined considerations for integrating guideline and QA methodology. We used the GIN-McMaster Guideline Development Checklist (https://cebgrade.mcmaster.ca/guidecheck.html) and created suggestions for an extension for guideline-QA integrated approaches on the basis of considerations identified in the preceding stages (feasibility study, systematic review, and evidence brief) [4].

### In person meetings and procedures

We invited seventeen participants based on their expertise in guideline or QA scheme development, or both (including quality indicator and performance measures development), to a three-day workshop. We identified the following relevant profiles: guideline and QA methodologists, IT technology specialists, epidemiologists, clinicians and a citizen advocate. Sixteen experts agreed to participate in the workshop. In addition, the four members of the steering group and two JRC researchers participated. Twelve of these participants are involved in the ECIBC activities. A full list of workshop participants is provided in the declarations section.

Following extensive preparations, the three-day workshop in June 2018 was structured around eight activities designed to move from a shared understanding of guideline and QA frameworks to the development of an integrated approach. Based on the findings of the evidence brief and review of the literature the first stage focused on agreeing on a common terminology for the integrated guideline and QA scheme methodological framework [18, 20]. The development of common language included review of the evidence brief and key background articles that were provided to participants. Next, participants discussed key questions, that we developed to frame the discussion around critical issues with a combined guideline and QA framework. Participants then reviewed the draft methodological framework and considered items that could be added as a QA extension to a guideline development checklist. Participants then presented comments on the key questions for discussion by the group.

Finally, the participants completed two online surveys – a survey relating to a risk analysis on the draft methodological framework, and a survey prioritising items for the checklist extension. To conclude, we had a discussion on the next steps following the workshop. We followed with telephone conferences to discuss drafts of the articles prepared and the overall approach until we reached consensus.

### Solutions to key questions/challenges

To guide the discussion on potential challenges, gaps, and solutions for integrating guideline and QA in the draft ECICC framework, we formulated 21 key questions and topics for consideration during the workshop. We based the key topics/questions (see Appendix 1) on the strengths, weaknesses, opportunities and threats (SWOT) analysis from the key informant interviews performed as part of the feasibility study for this project. The list was open to additional questions that emerged during the workshop.

### Risk analysis of draft framework

To obtain input on the draft framework, and to identify potential outstanding risks, we developed a risk analysis survey. Possible risks associated with the development of an integrated guideline and QA scheme were identified by workshop participants during the 3 days of meetings. They were continuously collected and collated in a survey using an online software (Survey Monkey, San Mateo, CA, USA) [21]. Each risk was assessed for its potential *severity* and *likelihood* on a scale of 1 to 5 (1 being lowest and 5 being highest). We then calculated a risk score using the multiplicative of the likelihood and impact for a total risk score from 1 to 25 (1 lowest and 25 highest). We calculated a mean risk for each possible item with all survey responses. We asked workshop participants to complete the survey during the final workshop session and displayed the results immediately for further discussion and clarification from the group.

### QA extension of GIN-McMaster guideline development checklist

The GIN-McMaster Guideline Development Checklist is used by numerous guideline developers to plan and facilitate the guideline development process [4]. To extend the checklist to inform the methodological framework of an integrated guideline and QA scheme, we collated the possible checklist items from participant suggestions over the three-day workshop and incorporated them into an online survey (Survey Monkey, San Mateo, CA, USA) [21]. We asked workshop participants to complete the survey at the conclusion of the workshop. The survey involved rating their agreement of 43 possible checklist item additions across 18 domains of the checklist. We asked participants to assess each item on a 7-point Likert scale from ‘strongly disagree’ to ‘strongly agree’. Ratings of agreement were converted into a numerical score from − 3 to + 3 and an average was calculated. A percentage agreement for each item was assessed dividing the average score by the maximum (+ 3) for each item. Agreement with checklist items, and additional suggested items will be used to inform the final checklist extension, which will be published separately.

### Thematic analysis

We brought together the feasibility study, findings of the systematic review, notes from the in-person workshop and the results of the two surveys on risk analysis and checklist extension as materials for qualitative thematic analysis. One reviewer (TP) drafted coding and themes reviewing survey results in Microsoft Excel (Microsoft Corp, WA, USA) and feasibility study transcripts and in-person workshop notes in Microsoft Word (Microsoft Corp, WA, USA), these were then reviewed and iteratively refined by a broader author group (HJS, ML, EP) and presented for feedback to the workshop participants and co-authors on this manuscript. Feedback on themes was incorporated into the final version presented.

## Results

### Feasibility study

The feasibility survey suggested a lack of methods and approaches for the integration of guideline groups and QA frameworks. We found that quality indicators may not relate directly to outcomes that guideline developers consider. We found that for ECICC an integrated framework should be feasible, but this will require engagement of QA experts during the framework development and close communication through the project.

### Thematic analysis

Seven key themes emerged as key considerations for integrating guideline and QA schemes: (1) evidence-based integrated guideline and QA frameworks are possible, (2) transparency should be used to clearly document the source and rationale for quality indicators, (3) intellectual and financial interests should be declared and managed appropriately, (4) selection processes and criteria for quality indicators need further refining (QIs), (5) clear guidance on retiring QIs should be included, (6) risks of an integrated guideline and QA Group can be mitigated, and (7) an extension of the GIN-McMaster Guideline Development Checklist should be undertaken to incorporate QA considerations. We present these themes in Table 1.

**Table 1:** Results of the thematic analysis

Theme	Description
(1) Evidence-based integrated guideline and QA frameworks	Integrated guideline and QA schemes should be based on the best available evidence (for QA schemes gray literature may be more relevant), usually synthesised and assessed in a systematic review. Evidence reviews should include not only the benefits but potential harms, and other considerations important for decision-making (e.g. GRADE Evidence to Decision (EtD) framework criteria).
(2) Transparency	The steps involved in linking evidence to guideline and quality assurance recommendations by an integrated framework should be clearly documented in a transparent manner.
(3) Declaration of interests and management of conflicts	Both financial and intellectual conflicts of interest for participants in an integrated guideline and QA scheme should be clearly declared and appropriately managed to limit interference in the process.
(4) Selection of QIs	Follow reporting standards on the selection of quality indicators from guideline recommendations [14]. Prioritise patient-important QIs that are measurable, feasible, cannot be easily manipulated and are sensitive to change. First select a small but sufficient number of candidate QIs for review. If QIs are not derived from guideline recommendations, clearly document their source and rationale.
(5) Retirement of QIs	A QI should be retired if, for example, it no longer addresses a quality gap, or it becomes associated with unintended consequences or harm emerges [14].
(6) Risks of integrated guideline and QA Group	We identified potential risks for a joint guideline and QA group, including challenges with group process, focusing on patient-important outcomes, unintended consequences, piloting of quality indicators, and achieving multi-stakeholder engagement. We concluded that the benefits of an integrate scheme outweighed the risks and that these risks would be manageable.
(7) Extension of guideline checklist to incorporate QA considerations	We added steps to the GIN-McMaster Guideline Development Checklist to incorporate unique QA considerations, such as searching for QIs, setting QA priorities, and whether expert subgroups within an integrated process are required to address QA.

#### Evidence-based integrated guideline and QA frameworks

We note that evidence, usually synthesised and assessed in a systematic review, should form the basis of guideline recommendations. Similarly, the QA group felt that QA schemes, should be generated based on a systematic review of the evidence on a relevant scheme and its effect. Workshop participants noted that a barrier to this is the lack of literature on the effect of QA schemes in many health domains. Nonetheless, participants felt that QA schemes and associated accreditation schemes, including those found in the gray literature, should be systematically reviewed prior to recommending them. The group further discussed that this review of the evidence for QA schemes should not only consider their ability to improve health outcomes, but also the unintended effects or harms, such as opportunistic behaviours by health providers or biases in reporting. Furthermore, feasibility, costs, and resource use for the implementation and administration of QA schemes should be considered, including if the QI are not the best measure or have unintended consequences.

We considered multiple planning steps for the integration of guideline and QA schemes into a common framework. Workshop participants recommended that an integrated framework should begin with a model on the health topic (e.g. logic model/analytical pathway/disease model/analytical PICO framework) that addresses the issue comprehensively from prevention to diagnosis to treatment. Within the analytical pathway, quality gaps should be identified for which quality indicators are deemed important to improve healthcare processes and outcomes.

Within the planning process for an integrated scheme we discussed that consideration should be given during the process of guideline development for the selection of possible QIs. It emerged that the GRADE EtD framework criteria lend themselves to highlighting when QA considerations are important [2, 22–24]. The group considered that one option could be a module in GRADE’s software GRADEpro (GRADEpro, Evidence Prime, Hamilton, ON, Canada) developed with features to enable parallel, integrated, or sequential QI development in relation to guideline development.

#### Transparency

We discussed the impact of guideline and QA integration on the transparency of recommendations. We found that to support the credibility and impact of an integrated guideline and QA scheme, clear documentation and rationale of the source and selection of QIs is important. There is an established process in guidelines, the GRADE EtD framework, to support transparency of guideline recommendations. Building from the GRADE EtD framework, the incorporation of QA scheme development would enable clear and transparent linkage of quality indicators to the literature evidence and guideline recommendations [2]. We considered that the implementation of a QA scheme may be considered an intervention in itself with potential impacts on health outcomes and the QA therefore be evaluated using an EtD like other guideline recommendations. EtDs consider criteria relating to the intervention and comparison including: how important is the problem, what are the desirable and undesirable health effects, what is the certainty of the evidence of effects, what resources are required, what is the certainty of the resources required, what is the cost-effectiveness, what are the impacts on health equity, is the intervention acceptable, and is the intervention feasible? Considering these factors for QA schemes, through the use of the EtD framework, has the potential to improve the evidence base that QA schemes are developed from and the transparent documentation of judgements as it has for guideline recommendations.

#### Declaration of interests and Management of Conflicts

We suggested that declarations of interests (DOI) and management of conflict of interest (COI) processes currently in place in many guideline groups should also apply to QA scheme groups. We note that in the ECIBC, the QA scheme development group underwent the same DOI process as the guideline development group. Workshop participants noted this is not always the case in QA scheme development. Individuals partaking in QA activities may similarly be affected by interests, whether they are financial or intellectual. We discussed the importance of disclosure and management of potential COIs for transparent and reliable QA schemes. We referenced the WHO guidelines for COIs and the nine core principles outlined in the GIN guidelines for COI [25, 26]. We discussed that these documents and principles should be considered and applied to an integrated guideline and QA scheme.

#### Selection or development of QIs

We elaborated on concepts and terminology relating to QA in another paper in this series [18]. A quality indicator refers to a construct used as a guide to monitor, evaluate, and improve the quality of services (e.g. Quality of Life). A performance measure refers to tools that quantify or describe measurable elements of practice performance (e.g. SF36). Finally, a performance indicator refers to measurable units of practice performance (e.g. score of 56 on SF36).

To discuss the selection or development of quality indicators within an integrated framework, we first considered that methods for deriving quality indicators from guidance should be described “clearly and in detail” [14]. We suggest that one or more quality indicators should initially be identified for each guideline recommendation, resulting in a list of potential ones. When the approach yields quality indicators that are not directly derived from a specific guideline recommendation, there should be special consideration and transparent reporting on the rationale for selection. We also suggested prioritisation of quality indicators that are valid and that cannot be manipulated by parties affected by them. In planning for integrated schemes, beyond the diversity of perspectives needed for guideline groups, we suggest that the integration of QA schemes also requires QA implementation stakeholders to be part of the consultation process.

For the development of quality indicators, we consider prioritising those outcomes, structures and processes that are relevant, which means important to patients, but which also implies a potential for improvement of health. Therefore, we suggested that the quality indicators should be measurable, feasible, and not be able to be manipulated by those that are affected by them. There was a minor difference in opinion about whether it should be taken into account if quality indicators are already in use and endorsed by professional societies. We discussed the importance of balancing presumed feasibility and acceptance of quality indicators in current use as well as their historical use for bench marking monitoring data, with a de novo assessment not currently in use for an unbiased judgement.

We discussed and referenced literature that described challenges with quality indicators. A major barrier to their performance is non-acceptance, and so we suggested that acceptability should be a key consideration of an integrated guideline and QA scheme group. Finally, we discussed the importance of selecting quality indicators that are sensitive to change, to avoid the ceiling or floor effect [27].

#### Retirement of QIs

We discussed at length the importance of retiring quality indicators and specifying the interval for updating QA schemes [14]. The group considered guidance from the literature on this topic [28]. The updating cycle for quality indicators may be linked to guideline updates. We considered that they may be retired when there is no longer a quality gap that requires measurement. Retirement or adaptation should be considered also if new evidence emerges that would change the recommendation on the use of a quality indicator (i.e. the identification of harm due to unintended consequences of the quality indicator).

#### Risks of an integrated guideline and QA group

The risk analysis pointed to potential threats associated with the integration of guideline and QA groups. Fifteen of the sixteen workshop participants responded to the risk analysis survey. The components of an integrated guideline and QA process that would constitute the highest risk to success if inadequately managed included: 1) group process and function for a joint guideline/QA group, 2) evaluating of accreditation based on QA schemes and the improvement of patient-important outcomes, 3) determining the unintended consequences of QIs, 4) piloting quality indicators within the context of a joint guideline/QA group, 5) achieving appropriate multi-stakeholder engagement for a joint guideline/QA group. The full risk analysis is available in appendix 2. We judged that mitigating these risks is feasible, and will be important to achieving a functioning integrated guideline and QA group.

#### Extension of the guideline development checklist to incorporate QA considerations

Finally, participants considered a list of draft checklist item additions and ranked them for consideration on final checklist additions. All sixteen workshop participants responded to this survey. The percent agreement for each item (ranked by descending agreement) ranged from 91.7 to 18.8%. The three items that had the highest agreement for inclusion were (*checklist category*): “Search for quality indicators and performance measures on the topic.” (*priority setting*); “Identify the perspective that is taken (population, individual, health system)” (*priority setting*); “Determine if subgroups on specific topics are required and how they will interact with the larger group.” (*guideline group membership*). The three items that had the lowest agreement were (*checklist category*): “Determine what accountability mechanisms will be developed for the quality indicators.” (*dissemination and implementation*); “Determine how the indicators will impact on accreditation and certification of organisations.” (*preparation for quality assurance and selection of quality indicators*); “Consider credibility of the institution in declaring what is known to individuals and what is not known at the time of declaration.” (*conflict of interest (COI) considerations*). The full checklist survey results will be published in a separate manuscript [29].

## Discussion

We conducted an extensive mixed-methods study to integrate guideline development and QA schemes under a common framework. The two worlds can learn from each other and this work identifies key considerations for both guidelines and QA schemes for the more effective integration to improve health outcomes. For the components to connect effectively, we recommend that a comprehensive model and framework will be necessary. The seven themes that emerged from the workshop will also be informative for those who intend to develop guideline-based QA measures. Performance measures have been part of guideline development in selected guidelines, and reporting standards have been developed for their development [14]. Guidelines, including the recent ECIBC, have utilized quality assurance to support recommendation implementation [30]. However, our work contributes novelty as existing efforts do not usually take an integrated approach so that evidence for recommendations informs to development. In the systematic review accompanying this article, three guidelines with quality assurance integrated to guideline recommendation evidence were identified [31–33]. However, this field requires further development and further research is required to address the risks and other challenges identified, to lead to a full and effective integration of these two worlds.

### Strengths and limitations

Strengths of our work include the detailed preparation and background material that informed the qualitative and quantitative aspects of our work. The experience with ECIBC, a recent high-profile guideline and QA project, provided a real-life example. Our process also began from an understanding of both guideline and QA methodological domains, and included experts from both of these worlds. We organised an effective set of interactions of participants from diverse organisational, professional, and geographic backgrounds.

Limitations of our presented approach include a focused group of experts within the guideline and QA fields, a topic focus on the specific case of a colorectal cancer guideline, and outstanding questions and uncertainties that exist for integrated QA and guideline schemes. Although we were strategic in selecting participants from many disciplines, background, and geographic regions, one weakness of our present work is that we have not achieved full representation of global efforts in guideline development and QA. The reason for this was to maintain a manageable group size. This, combined with unanswered questions on the particulars of integrating guideline and QA schemes, leaves significant future methodological research in this domain to plan and assess the implementation of integrated frameworks across topics in health in different settings and for different purposes. Our effort focused on colorectal cancer as an example; efforts in other health domains may have unique considerations, though we believe the general principles are generalisable and applicable to other health domains. The resources and logistics required for the integration of guideline and QA efforts are unknown and will need to be assessed and this will affect the feasibility of implementing of an integrated framework in different resource settings and at different levels.

### Implications for practice and research

There are several implications for practice in guideline and QA scheme development, health research, and policy. We have concluded that an integrated framework for guideline and QA is feasible, and despite the challenges of integration would provide numerous benefits to improve the linkage of the process and impact of both. The seven themes that emerged will require operationalisation, integration, adoption and updating through various means, including software solutions. For future guideline or QA schemes, both should consider implications for the other and whether an integrated framework is possible for the given topic and our seven key findings will help with that. If integrated development is not deemed to be possible, considerations should be given to subsequent integration and documentation of the two processes.

Through the workshop and activities that followed, we identified next steps and a research agenda for the integration of guideline and QA schemes. The workshop participants indicated an interest in furthering the collaboration to the research agenda of integrating guideline development and QA. Future activities we identified included the systematic review and terminology paper as part of the present series, an update to the GIN reporting criteria checklist, criteria for retiring QIs, consideration of DOI/COI in QA, and addressing implementation/evaluation of integrated guideline and QA frameworks. Finally, the findings we present here will now be integrated into the final methodological framework for implementation in the ECICC.

## Conclusions

This article presents the findings of a mixed-methods approach centering on the development of a methodological framework for integrating the guideline and QA schemes of the forthcoming ECICC. We present seven key themes resulting from the iterative process. We conclude that the integration of guideline and QA is feasible in this context and presents clear benefits and that the challenges identified are surmountable. Of note extensive methodological work to more effectively integrate and evaluate guidelines and QA schemes is required. In particular, attention to the use of evidence and transparency of integrated processes is critical. We have presented possible solutions for many of the challenges identified. The findings will serve as a roadmap to inform the future work of developing an integrated guideline and QA scheme framework and, in the meantime, serve as considerations for practical guideline and QA development groups. We will consider these for the forthcoming ECICC, which will be a large-scale integrated scheme and serve to advance research into this approach. Further research will continue to augment the capacity for integration of guideline and QA schemes to bring these two critical worlds closer together, to advance both health guidance and QA to ultimately improve health.

## Data Availability

Upon request.

## Appendix 1

**Table 2:** Key Topics/Questions to Inform Workshop

Key topic/question (topics were content domains for exploration and questions specific answerable questions)	
1	What is the starting point for QI development? E.g., are the recommendations leading, or does one decide beforehand for which care aspects one wants to develop QI (see draft frameworks sections 1 and 2 in the GIN-McMaster checklist – draft framework)? What if there is only low certainty evidence for these aspects? (key topic)
2	We will need a GRADEpro QI development module: what would be the design? A module that runs parallel to the recommendation development, or integrated with the Evidence to Decision part? Or in addition to EtD? This is a fundamental question! (key question and topic)
3	How to move from recommendation to QI and from QI to performance measure? See Kahn 2014 and Shekelle 2013. When is a recommendation suitable for a QI, with attributes of good quality indicators in mind, and ‘translatability’, can the quality of evidence and EtD factors be of any help here? Which are applicable to QI development? (key topic)
4	How can GRADE help to make the process of QI development more transparent and explicit? (key question)
5	Based on what we learned and read, is there anything that should be added to the GIN reporting checklist for quality indicators and performance measures (Nothacker et al. – workbook documents). (key question)
6	Colorectal cancer screening can be done with many different approaches and sequence of tests and treatments. They should be evaluated in multiple intervention comparisons (of multiple tests and treatments). What is the impact of that on quality indicators (e.g. do we need quality indicators that can be applied to all test-treatment strategies)? (key question)
7	Does conflict of interest management differ in QA from developing the recommendations? (key question and key topic)
8	QI can assess structures, processes and outcomes of health care. Good outcome measures do not necessarily stand for good quality of care, while the application of process and structural indicators in daily practice can lead to better outcome measures. Impactful process and structure outcomes lead to better outcomes, but this should be underpinned by evidence. How can we include that in the framework, or is that somehow covered by certainty in the evidence (e.g. indirectness)? (key topic)
9	The involvement of professional societies, accreditation and certification bodies seems indispensable for implementation of guidelines and quality assurance. Are technical observers and consultants (non-voting on recommendations) appropriate to make informed decisions and here context? How and when should they be involved? (key question)
10	QI may relate to a) patient important outcomes addressed in recommendations; b) process issues (e.g. number of lost specimens in the laboratory pathway); c) diagnosis (tests correctly read by pathologists); d) informed choice (adequate use of processes of decision-making) that consider patient values and preferences. Which are the other domains that a joint guideline/QA needs to consider? Explain your considerations please. (key question and key topic)
11	We propose one working group instead of GDG and QASDG: what are the member criteria (expertise, which disciplines)? What are the training needs? (key question and key topic)
12	Regarding outcomes in quality assurance and quality indicators: do we need or want to define the clinical question/PICO/outcomes with QI development in mind? E.g. surrogate outcomes are often more feasible to measure than direct patient relevant outcomes, especially those that take longer time to occur. However, surrogate outcomes often lower the certainty in the evidence. How to rate the importance of outcomes – should quality indicators be determining the importance of outcomes (do outcomes become people important if they can be measured as performance indicators?)? (key question and key topic)
13	How would you expand the monitoring and evaluation section of the GRADE evidence to decision frameworks based on the lessons learned and the draft framework (click here for a description of the EtD templates)? (key question and key topic)
14	How should we integrate all QA aspects: quality indicators, performance indicators, requirements, certification, a quality assurance scheme? Quality indicators and performance measures determine the requirements for certification. Which aspects are logically related to development of recommendations and which aspects need a different route? (key topic)
15	How to assess certainty of the evidence of QI? Do we need such an assessment? E.g. by use of instruments for risk of bias appraisal of QI (QUALIFY or AIRE) extended to a full assessment? (key question)
16	How can we assess and express the potential benefit of proposed QI/performance measure (being one of the criteria for a good QI)? (key question and key topic)
17	Involvement of patients in QI development: how and in which stages? Results of the study by Schleedoorn 2016 et al. support the fact that there is poor correlation between patients’ and professionals’ perceptions regarding quality of care. Their set of key recommendations would have been different if only medical professionals were involved in the selection procedure. (key question)
18	For guideline and quality assurance development processes, expertise in many areas is required. How can groups function efficiently, how can subgroups of a panel interact with the main panel? E.g., should groups work separately to develop recommendations and quality indicators and ask a core panel to agree or vote on them? How can experts be involved on an ad hoc basis without being a full member of a panel? (key topic)
19	Should we provide guidance on how many QI to develop? Studies show an average number of 20–50 quality indicators per condition, but ideally the development of quality indicators results in a compact set of ≤5 indicators per clinical area (key question)
20	Reflecting on the table below, should quality indicators or performance indicators be based only on strong recommendations (see implications of strong and conditional recommendations)? How can we relate certainty of the evidence and strength of recommendations to QI development? What is the rationale (see Kahn, 2014)? How to best implement conditional recommendations as PI? (key topic)
21	When should modelling be integrated in guideline development? (Key question)
22	Should there be criteria for “retirement” of a guideline-based quality indicator?

## Appendix 2

### Risk analysis

The risk analysis included a review of key challenges and was focused on the external experts. It included a rating of the likelihood and severity of risks related to the items we offered for review. Fifteen of the sixteen external participants responded to the survey for a response rate of 94%. The range in risk scores was from 6.6 to 12.4 (scale from 1 to 25). The item identified as the highest likelihood (to happen) was “How to consider performance measures that do not come from a specific recommendation.”; the item rated as the lowest likelihood was “Management of conflicts of interest relating to guideline and quality assurance development”. The item rated as having the highest impact was “Group process and function for joint guideline/QA participants” and the item rated as the lowest impact was “Developing criteria relating to when to ‘retire’ a quality indicator”. Not unexpectedly, the item rated overall as the highest risk was “Group process and function for joint guideline/QA participants”. The item rated overall as the lowest risk was “Developing criteria relating to when to ‘retire’ a quality indicator”.

**Table 3:** Risk Assessment for the Integration of Quality Assurance and Guideline Development

Item	Risk (/25)
Group process and function for joint guideline/QA participants.	12.4
Evaluating whether accreditation based on performance measures improves patient-important outcomes.	11.8
Determining the unintended consequences of quality indicators.	11.7
Piloting quality indicators within the context of a joint guideline/QA group.	11.3
Achieving appropriate multi-stakeholder engagement for a joint guideline/QA group.	11.0
Identifying valid surrogate quality indicators that are related to patient-important outcomes.	11.0
How to consider performance measures that do not come from a specific recommendation.	10.9
Determining what the starting point is for QI development (e.g. risk that recommendations are leading).	10.9
Determining how to integrate quality indicators and evidence-to-decision frameworks.	10.4
Determining how subgroups will inform QI development in the context of a joint guideline/QA group.	9.8
Selecting the right number of QIs (not too many or few).	9.2
Distinguishing/prioritising between individual/patient and population-oriented quality indicators.	8.6
Considering the use of modelling evidence in a joint guideline/QA group.	8.6
Management of conflicts of interest relating to guideline and quality assurance development.	8.4
Conducting a prioritisation exercise for quality indicators.	8.4
Developing consensus on the criteria that should be included as monitoring and evaluation considerations in an EtD.	7.1
Developing criteria relating to when to ‘retire’ a quality indicator.	6.6

## Abbreviations

COI: Conflict of Interest
ECIBC: European Commission Initiative on Breast Cancer
ECICC: European Commission Initiative on Colorectal Cancer
EtD: Evidence to Decision framework
GIN: Guidelines International Network
GRADE: The Grading of Recommendations Assessment, Development and Evaluation
ISQua: International Society for Quality in Healthcare
JRC: Joint Research Centre
PICO: Population, Intervention, Comparison, Outcome
QA: Quality Improvement
SWOT: Strengths, Weaknesses, Opportunities, Threats
